# A Naloxone Best Practice Advisory That Failed to Signal: A Real‑World Evaluation of an Emergency Department

**DOI:** 10.7759/cureus.103760

**Published:** 2026-02-17

**Authors:** Aretha D Miller

**Affiliations:** 1 Nursing, Swedish Medical Center, Seattle, USA

**Keywords:** alert fatigue, best practice advisory, clinical decision support, emergency department, harm reduction, health equity, naloxone

## Abstract

Background: Naloxone distribution is a key harm-reduction strategy, yet implementation in emergency departments (EDs) remains inconsistent. Best Practice Advisories (BPAs) are intended to provide timely, actionable prompts, but high alert burden can diminish their effectiveness through alert fatigue. Existing evidence largely reflects academic medical centers and focuses on naloxone prescribing rather than the distribution of take-home naloxone (THN) kits. Little is known about how naloxone-related BPAs perform in community EDs, where workflows and alert-fatigue dynamics differ.

Methodology: We conducted a retrospective cross-sectional study in a suburban, non-academic ED from January 2022 to January 2024. After exclusions, 10,313 encounters with complete BPA and naloxone documentation were analyzed. Six naloxone-related BPA elements were extracted from audit logs and evaluated alongside demographic and clinical variables. Multicollinearity among BPA components was addressed using variance inflation factor (VIF)-guided pruning. Class imbalance was mitigated using SMOTE-Tomek and class weights. Logistic regression models were developed using an 80/20 train-test split, with performance assessed using area under the curve (AUC), accuracy, and sensitivity analyses.

Results: Among encounters in which a naloxone-related BPA fired, clinicians overrode 25.6% of alerts and acknowledged 74.4%; however, acknowledgment translated into THN distribution in only 36.7% of encounters. This highlights a substantial gap between alert visibility and clinical action. Furthermore, BPA-related variables contributed minimal predictive signal. In adjusted models, the Emergency Severity Index (ESI) was the only significant predictor, with lower acuity associated with decreased odds of naloxone distribution. Race, ethnicity, sex, age, and all BPA-related variables were not significant predictors. The final model demonstrated moderate discrimination (AUC ≈ 0.77) with stable performance across original and SMOTE-balanced datasets. Subgroup analyses showed no evidence that BPAs improved equity in naloxone distribution.

Conclusions: Naloxone BPAs in this community ED did not function as meaningful clinical signals. Although most alerts were acknowledged, they did not influence the distribution of THN kits and added minimal predictive value. Naloxone distribution reflected clinical cues, particularly acuity and opioid-related risk indicators, rather than BPA prompts. Redesign is needed to improve the timing, targeting, and relevance of naloxone-related clinical decision support (CDS). Future harm-reduction strategies should prioritize workflow-integrated, actionable CDS rather than broad, interruptive alerts that blend into background noise.

## Introduction

Background/rationale

Clinical decision support (CDS) systems are essential digital health tools that enhance clinical decision-making by providing clinicians with evidence-based, patient-specific information at the point of care. These systems have evolved from simple rule-based alerts to more sophisticated, AI-enabled tools capable of integrating real-time electronic health record (EHR) data to guide clinical action [1-3].

Among the most widely used CDS mechanisms is the Best Practice Advisory (BPA), an automated, interruptive notification embedded within the EHR that prompts clinicians to review key information and consider an evidence-aligned action [2]. The CDS Five Rights framework emphasizes that effective CDS must deliver the right information to the right person, in the right format, through the right channel, and at the right point in the workflow [3]. BPAs are designed with this principle in mind, functioning as brief interruptions intended to momentarily pause workflow so clinicians can reassess critical information before proceeding [2].

However, BPAs exist within a broader ecosystem of interruptive alerts that share inherent trade-offs. While such alerts can improve adherence to evidence-based practices, they also impose cognitive and workflow burdens and contribute to alert fatigue, a well-documented phenomenon in which clinicians become desensitized to frequent alerts, leading to increased override rates and reduced responsiveness [4,5]. Over time, even clinically appropriate alerts may be dismissed reflexively, limiting their intended impact and potentially compromising patient safety [4].

Despite their widespread use, relatively little is known about how specific BPAs perform in real-world clinical environments. Existing literature focuses largely on BPA design principles or optimization strategies, with fewer studies evaluating whether a BPA meaningfully influences clinician behavior or patient care when deployed in routine practice [2,6]. Key questions remain regarding how often BPAs fire, how frequently they lead to the desired clinical action, and whether their performance varies across patient subgroups or clinical contexts [2,6,7].

This study evaluates the performance of a naloxone-related BPA within a large Emergency Department (ED) setting. The analysis examines BPA firing patterns, clinician responses, and associations with the distribution of take-home naloxone (THN), an ED-based harm-reduction program in which at-risk patients receive a naloxone kit and brief education to prevent fatal opioid overdose [8].

Objective

This study evaluated whether the activity of six naloxone BPAs in an ED significantly predicted the distribution of THN kits or functioned merely as background noise devoid of clinical utility.

Hypothesis

The implementation of BPAs has no statistically significant impact on the distribution of THN kits in an ED.

## Materials and methods

Study design and setting

This retrospective cross-sectional study was designed and reported in accordance with the STROBE guidelines [9]. It analyzed ED encounters occurring between January 1, 2022, and January 1, 2024, at a suburban, non-academic medical center with a 35-bed ED. Institutional Review Board approval was obtained in October 2024, and data collection was completed in February 2025.

Participants

All ED encounters during the study period were screened for eligibility. Encounters were included if they contained complete EHR documentation for all BPA variables and naloxone distribution status. Encounters were excluded when key BPA fields, naloxone outcome data, or essential demographic or clinical variables were missing, or when records were duplicated or corrupted. All inclusion and exclusion criteria were defined a priori.

Data sources and measurement

All variables were extracted from the hospital’s EHR. Naloxone distribution data were obtained from the medication administration record and discharge documentation. BPA-related variables (OPA Record Name, Override Reason, Reason for Visit/Call, Trigger Point, User Follow-up Action, and Warning Name) were sourced from the BPA audit log, which automatically records alert firing events and provider responses using standardized system-generated fields.

Demographic and clinical variables, including age, sex, race, ethnicity, language, ICD-10 diagnoses, triage acuity, chief complaint, substance use history, and medication exposure, were obtained from structured EHR fields routinely completed during ED intake and clinical documentation.

Naloxone BPAs commonly fire at ED discharge, aligning with evidence that EDs frequently provide THN during the discharge process as part of harm-reduction efforts. Prior evaluations of ED naloxone programs show that naloxone is most often dispensed at the point of discharge, reinforcing the rationale for CDS tools to trigger at this workflow step [8]. All encounters were documented within the same EHR system using consistent workflows and BPA logic, ensuring comparability across encounters.

Variables

Primary Outcome

Naloxone distribution was defined as a binary outcome indicating whether a THN kit was dispensed during an ED encounter (1 = yes, 0 = no).

Primary Exposure Variables: BPA Features

Six BPA-related variables were extracted to characterize alert activity: OPA Record Name, Override Reason, Reason for Visit/Call, Trigger Point, User Follow-up Action, and Warning Name. All BPA variables were originally stored as True/False fields and were converted to numeric format (1 = true, 0 = false). Non-convertible values were assigned as missing and imputed as 0 to preserve the binary structure.

Predictor Variables

Predictors included demographic characteristics (age, sex, race, ethnicity, language), clinical characteristics (opioid-related ICD-10 diagnoses, IV drug use history, unknown illicit drug use, comorbidities, triage acuity, and chief complaint), and encounter-level variables (employment status, readmission history, and medication exposure such as morphine milligram equivalents per day (MME/day) and morphine equivalent daily dose (MEDD) groups).

Potential Confounders

Potential confounders were identified using prior literature, chi-square tests, and Cramér’s V rankings. Variables retained for adjustment included age, sex, opioid-related diagnoses, substance use history, triage acuity, chief complaint category, and medication exposure.

Bias

Selection bias was minimized by including all eligible encounters within the study period and excluding only those with incomplete BPA or naloxone documentation. Information bias was reduced by relying exclusively on structured EHR fields and standardized BPA audit logs. Misclassification bias was addressed through uniform binary conversion of BPA variables and systematic imputation of non-convertible values. Confounding was managed through a combination of a priori identification of clinically relevant variables and statistical screening.

Study Size

Participant flow through the study is summarized in Table 1. A total of 12,272 ED encounters in which at least one naloxone-related BPA fired between January 1, 2022, and January 1, 2024, were identified. After screening for completeness of BPA documentation, naloxone distribution status, and essential demographic and clinical variables, 1,959 encounters were excluded due to missing or non-usable data or duplicate or corrupted EHR entries. The final analytic dataset consisted of 10,313 BPA-triggered encounters with complete information available for all planned analyses. Since the study used a census of all eligible records, no a priori sample size calculation was performed. 

**Table 1:** Participant Flow and Study Size Summary of Emergency Department (ED) encounters screened for eligibility, exclusions due to incomplete documentation or unusable records, and the final analytic dataset used for all analyses. Post-hoc detectable effect size reflects the minimum standardized difference detectable with α = 0.05 and 80% power given the observed sample size.

Stage	Value
Total eligible Emergency Department encounters	12,272
Excluded encounters (incomplete BPA or naloxone documentation; duplicates; unusable records)	1,959
Final analytic dataset	10,313
Post-hoc detectable effect size (Cohen’s d)	0.06

A post-hoc power analysis using α = 0.05 and 80% power, based on the observed sample size and an approximate 30/70 distribution between minoritized and reference groups, indicated that the study was powered to detect a minimum standardized effect size of approximately 0.06 (Cohen’s d).

Quantitative Variables

Age, medication exposure (e.g., MME/day and MEDD), and other continuous variables were retained in their original form and standardized using z-score scaling. No arbitrary cut points were introduced. Categorical variables with multiple levels were transformed using one-hot encoding to preserve category granularity without imposing an ordinal structure. High-cardinality categorical variables were retained in their encoded form.

Missing Data

Encounters missing essential BPA information or naloxone distribution status were excluded. BPA fields that could not be converted to numeric values were coded as missing and imputed as zero to preserve a consistent binary structure. For demographic and clinical variables, missing values were handled after one-hot encoding, where absent categories were represented as zeros, ensuring a complete predictor matrix without introducing artificial cut points. All variables were assessed for missingness prior to model fitting.

Analytical methods accounting for the sampling strategy

The study used a census of all eligible ED encounters rather than a probability-based sampling design; therefore, no weighting or survey-design adjustments were required. Analyses incorporated all eligible encounters, with missing data handled as described above. Logistic regression models were fitted using the full analytic dataset.

Statistical methods

Descriptive statistics characterized the demographic and clinical features of the cohort. Chi-square tests with Cramér’s V were used to evaluate associations among categorical variables and guide preliminary feature selection. Continuous predictors were standardized using z-score scaling. Multicollinearity was assessed using VIF, and variables exceeding the threshold were removed. Confounders were identified through a combination of a priori clinical relevance and statistical screening. Ordinary Least Squares diagnostics demonstrated deterministic multicollinearity among BPA component fields, as shown in Table 2. 

**Table 2:** Multivariable Logistic Regression Predicting Override of Naloxone BPA The multivariable logistic regression model examined the influence of demographic, clinical, and workflow-level variables on naloxone BPA override behavior. The results demonstrate that override patterns were remarkably uniform across nearly all categories. Most predictors, including patient demographics and BPA-specific fields, yielded odds ratios near 1.0, with confidence intervals crossing unity, indicating no statistically significant association with override behavior. The Emergency Severity Index (ESI) was the sole significant predictor (OR = 0.730; 95% CI (0.700, 0.760)), with higher ESI values (lower clinical acuity) associated with significantly reduced odds of overriding the BPA. Predictor scope: The model includes demographic, clinical (Length of Stay Days (los_days), Morphine Milligram Equivalents (mmeday), Home IV Drug Use Risk (homeivdu_risk), and workflow-specific (e.g., trigger point) predictors. Anonymity: To ensure provider privacy, all clinician names have been replaced with alphabetical aliases (Alpha, Beta, Kappa). Interpretation: An odds ratio greater than 1 indicates increased odds of override; an odds ratio less than 1 indicates decreased odds. Significance: Bolding denotes statistical significance. BPA: Best Practice Advisory; CI: confidence interval; ESI: Emergency Severity Index; OR: odds ratio.

Predictor	Odds Ratio	95% CI	Coefficient
los_days	1.009	0.967–1.053	0.00917
mmeday	1.007	0.964–1.052	0.00710
age	1.004	0.958–1.051	0.00363
user_Alpha (Alias)	1.003	0.771–1.306	0.00318
user_Beta (Alias)	1.002	0.703–1.427	0.00190
… additional clinician fixed‑effects …	—	—	—
user_Kappa (Alias)	0.991	0.795–1.235	–0.00910
homeivdu_risk	0.985	0.916–1.059	–0.01527
opa_record_name_ED OP NALOXONE NEEDED FOR AT RISK…	0.955	0.844–1.080	–0.04609
trigger_point_Sign Orders	0.948	0.841–1.068	–0.05363
esi	0.730	0.700–0.760	–0.31494

Multiple logistic regression models were used to examine associations between BPA-related predictors and naloxone distribution. The dataset was randomly partitioned into training (80%) and testing (20%) subsets. Class imbalance was addressed using SMOTE-Tomek resampling and adjusted class weights. Model performance was evaluated using pseudo R-squared values, the area under the receiver operating characteristic curve (AUC), and ROC curve visualization. Statistical significance was defined as α = 0.05.

Subgroups and interaction analyses

Subgroup analyses evaluated whether associations between BPA-related predictors and naloxone distribution differed across demographic and clinical groups. Stratified descriptive comparisons were used to identify potential variation in patterns of association. Interaction terms between key covariates (e.g., chief complaint category, substance use history) and BPA-related predictors were examined in exploratory logistic regression models using the VIF-screened predictor set.

Sensitivity analyses

Sensitivity analyses evaluated the robustness of findings across multiple modeling decisions. Logistic regression models were re-estimated using the full VIF-screened predictor set and compared with models restricted to predictors that remained statistically significant in the primary model. Models were also re-run without SMOTE-Tomek resampling to assess the impact of class imbalance correction. Additional robustness checks varied the Cramér’s V and VIF thresholds used during preliminary screening and multicollinearity assessment. Across all analyses, the direction and significance of primary associations remained consistent.

## Results

Participant flow

A total of 12,272 ED encounters in which at least one naloxone-related BPA fired were identified between January 1, 2022, and January 1, 2024. All encounters were screened for completeness of BPA documentation and naloxone distribution status. During screening, 1,959 encounters were excluded due to incomplete BPA fields, missing naloxone status, missing demographic/clinical variables, or duplicate or corrupted entries. The remaining 10,313 BPA-triggered encounters contained complete information and were included in the analytic dataset. All 10,313 encounters were retained for descriptive analyses, model development, and subgroup evaluations. No patients declined participation because the study used de-identified retrospective EHR data.

Descriptive data

The final analytic dataset included 10,313 ED encounters with complete BPA and naloxone documentation. Patients represented a broad age range, with the highest encounter volume occurring among older adults. Both males and females were represented across all age categories.

Most patients identified as White and non-Hispanic, and English was the predominant primary language, although several other languages were represented in smaller numbers. Social characteristics varied, with most patients documented as single and employment status distributed across unemployed, retired, and full-time employee groups.

Clinical characteristics also demonstrated substantial heterogeneity. Common presenting complaints included abdominal pain, chest pain, falls, shortness of breath, back pain, and accidental overdose. Frequently documented ED diagnoses included abdominal pain, lumbar radiculopathy, chest pain, ureteral stone, and opioid-related conditions. Substance use history and alcohol use patterns varied across the cohort. Medical comorbidities such as hypertension, diabetes, depression, chronic lung disease, and HIV/AIDS were commonly observed.

Encounter-level characteristics reflected a wide distribution of visit dates. A subset of patients had received naloxone within the prior two years. All variables included in multivariable models were complete after exclusion of encounters with missing BPA or naloxone documentation and application of standardized preprocessing procedures.

Outcome data

THN was distributed in 36.7% of encounters. Distribution frequencies varied across demographic, clinical, and encounter-level characteristics, with higher proportions observed among patients presenting with opioid-related complaints, those with documented substance use histories, and those with prior naloxone administration within the past two years.

After preprocessing to remove duplicate encounters and those with incomplete BPA or naloxone documentation, 10,313 encounters with complete BPA firing information were available for analysis. All outcomes were assessed at the index encounter; no longitudinal follow-up was conducted.

Main results

In the adjusted logistic regression models that accounted for demographic, clinical, and encounter-level covariates, race was not significantly associated with naloxone distribution. No racial or ethnic group demonstrated higher or lower adjusted odds of receiving a naloxone take-home kit compared with the reference category. These findings were consistent across sensitivity analyses and subgroup evaluations.

Adjusted predicted probabilities by race/ethnicity

Adjusted predicted probabilities of naloxone distribution did not differ meaningfully across racial or ethnic groups. In fully adjusted models, race and ethnicity were not significant predictors of naloxone distribution, and no evidence of effect modification by BPA-related variables was observed. Predicted probabilities remained stable across demographic subgroups, indicating that clinician responses to naloxone-related BPAs were consistent regardless of patient race or ethnicity.

Other predictors

**Figure 1:**
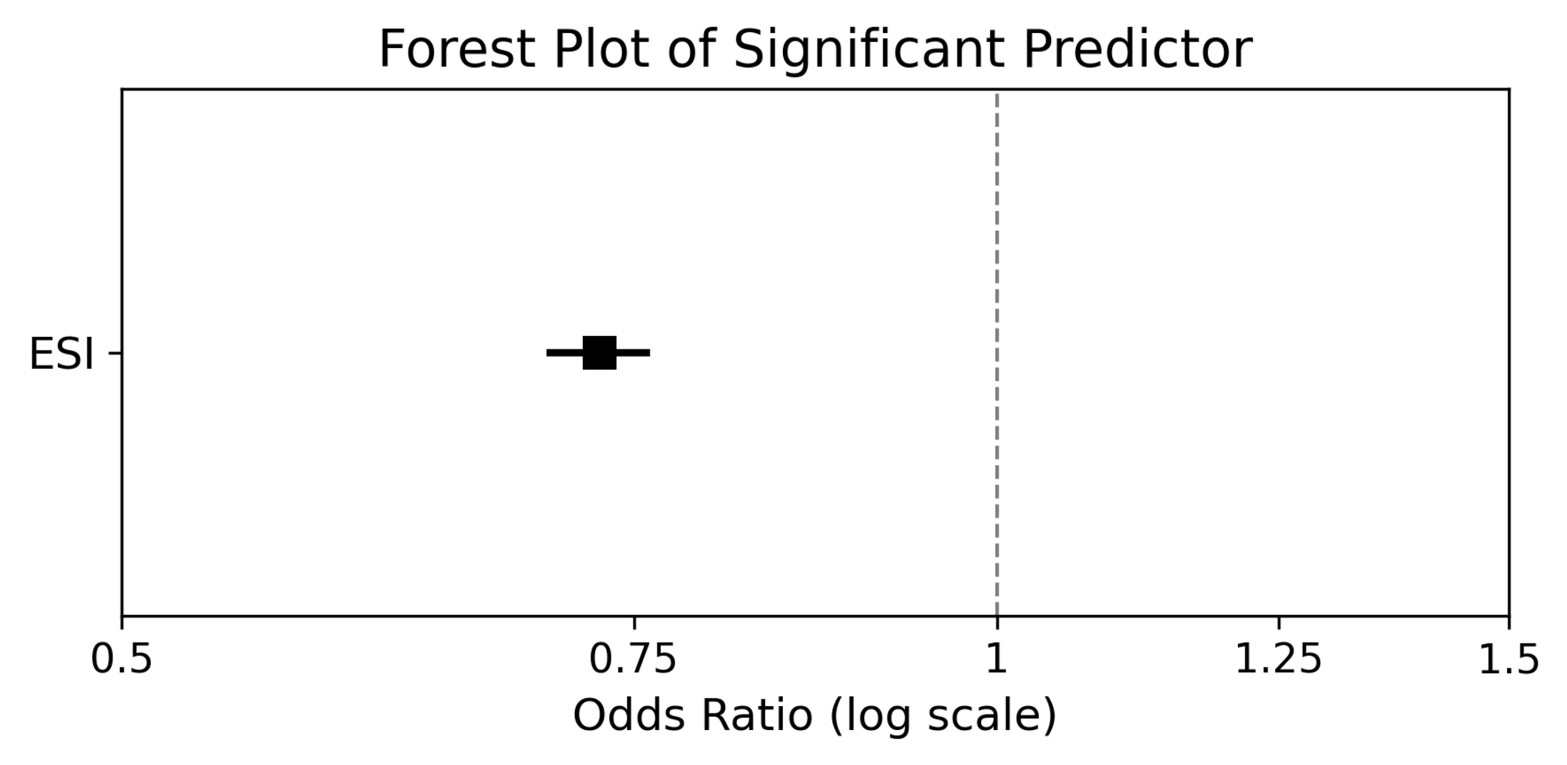
Forest Plot of Significant Predictor Forest Plot of the model’s only significant predictor, showing the adjusted odds ratio and 95% confidence interval on a log scale. The point estimate and horizontal confidence bar illustrate both the direction and precision of the association.

In adjusted models, neither sex nor age demonstrated clinically meaningful associations with naloxone distribution. The small effect estimates observed for these variables were attenuated after full adjustment and were not consistent across sensitivity analyses. No demographic or clinical covariates showed strong or stable associations with naloxone distribution beyond expected patterns related to presenting complaint and substance use history. The forest plot in Figure 1 displays the adjusted odds ratio and 95% confidence interval for the model's only significant predictor, illustrating the direction and precision of its association. 

Effect of BPA firing

In adjusted models, BPA firing was not a meaningful predictor of naloxone distribution. Although an early model iteration showed a small, statistically significant association, this effect was not stable after resolving multicollinearity and applying the final VIF-screened predictor set. Across all sensitivity analyses, BPA-related variables demonstrated no substantive predictive value, consistent with the uniform clinician response patterns observed in descriptive analyses.

Model performance

**Figure 2:**
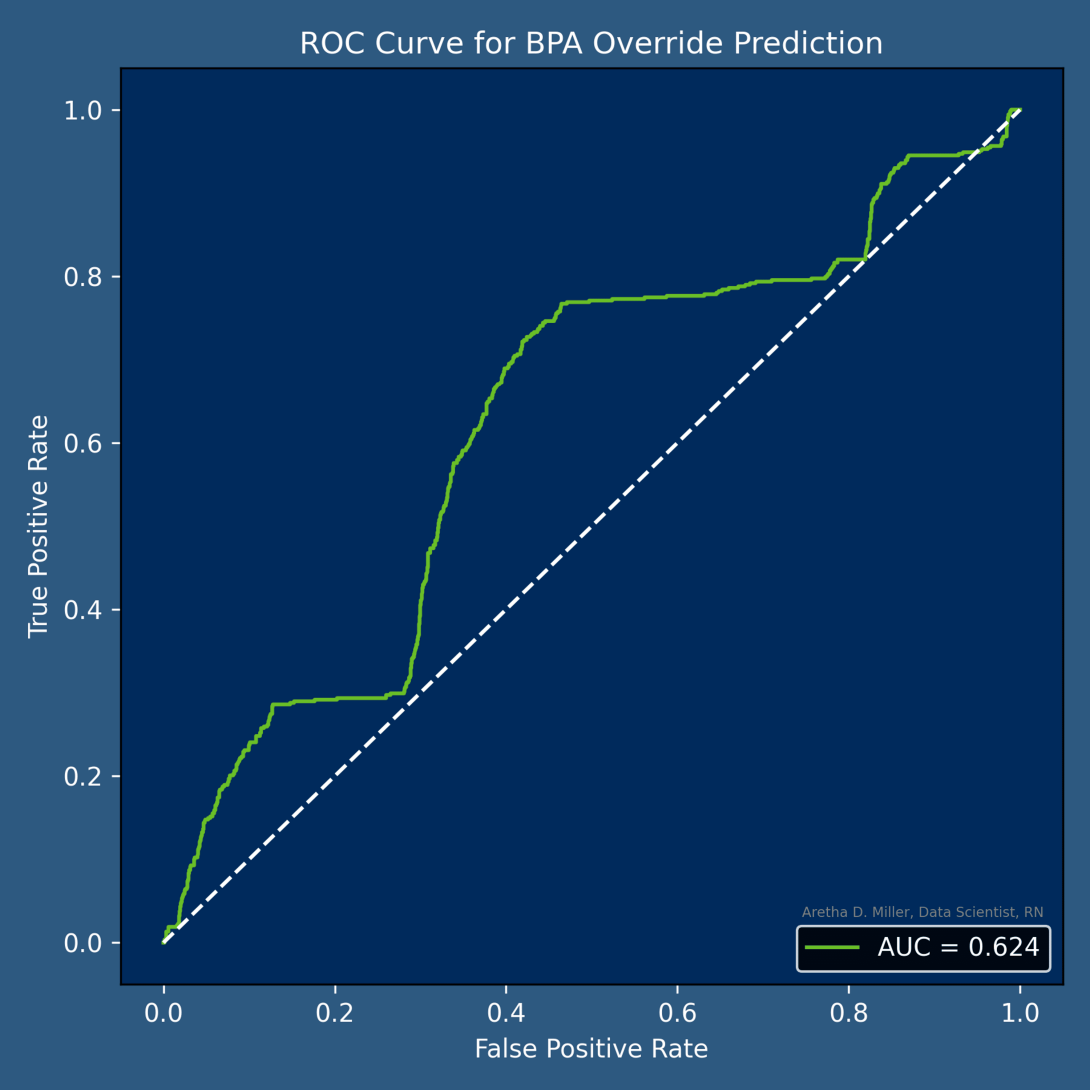
ROC Curve. BPA-Based Prediction of Naloxone Distribution ROC curve demonstrating model discrimination for predicting Naloxone take‑home kit distribution using BPA‑related, demographic, clinical, and encounter‑level predictors. The final model achieved an AUC of 0.624, indicating limited discrimination. ROC: receiver operating characteristic, BPA: best practice advisory, AUC: area under the curve.

The original logistic regression model showed limited discrimination (AUC = 0.624), consistent with performance observed in both the original and synthetic minority over-sampling technique (SMOTE)-balanced datasets. Model discrimination for the BPA override prediction is presented in Figure 2.

**Figure 3:**
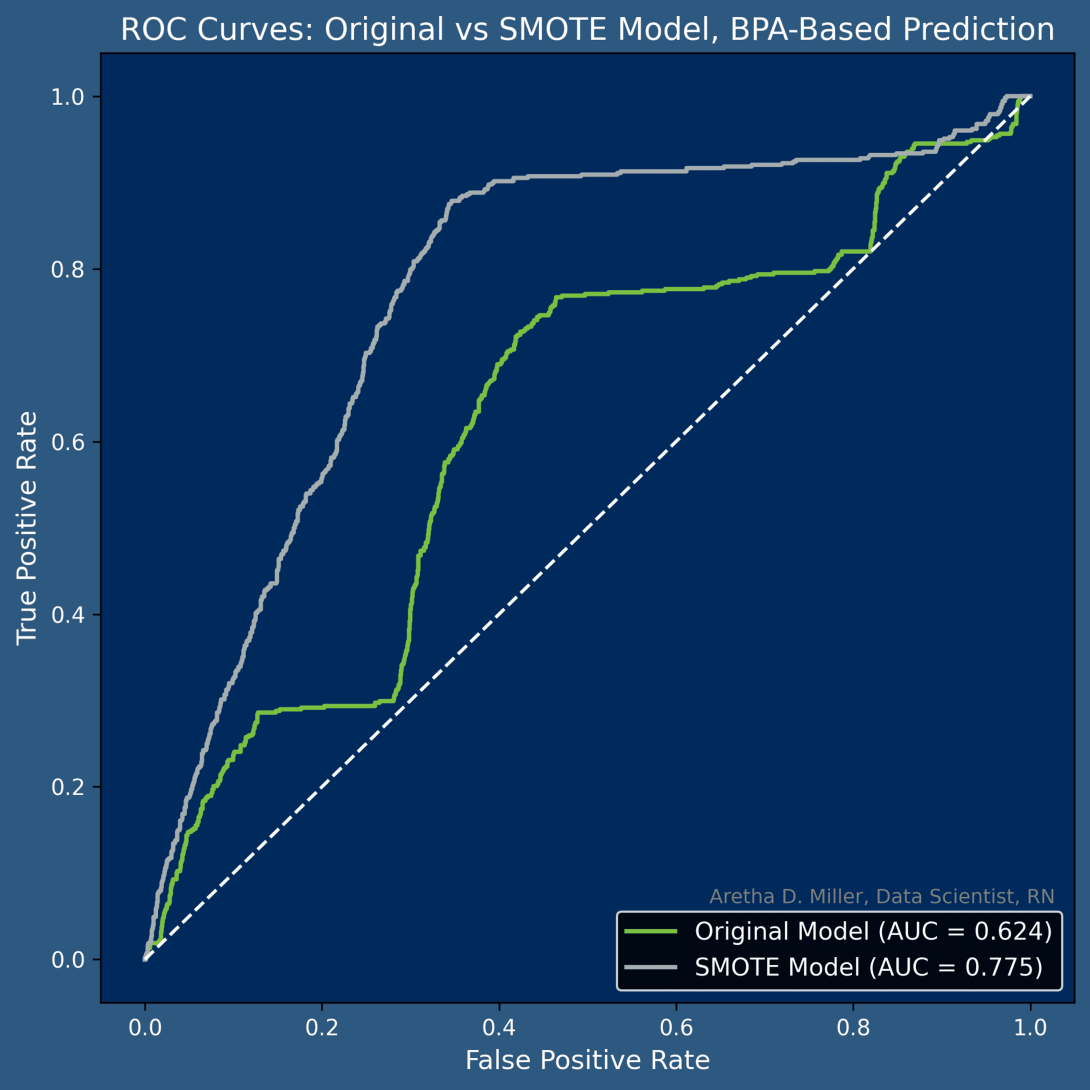
ROC Curve. Original vs Smote Model, BPA-Based Prediction This figure compares the discrimination performance of two logistic regression models, one trained on the original imbalanced dataset and one trained on a SMOTE‑balanced dataset using BPA‑related variables to predict the distribution of Naloxone take-home kits. The SMOTE model shows a modest improvement in the ROC curve and AUC compared with the original model, but both curves remain relatively close to the diagonal reference line. This indicates that BPA‑based predictors contain limited signal and have low ability to distinguish encounters with versus without Naloxone distribution. ROC: receiver operating characteristic, SMOTE: synthetic minority over-sampling technique, BPA: best practice advisory, AUC: area under the curve.

Model accuracy and class-specific precision and recall reflected the underlying class imbalance, with higher performance for the majority class and lower but stable performance for the naloxone distribution class. Figure 3 compares the ROC curves for the original and SMOTE-balanced models, demonstrating modest improvement in discrimination after resampling. Overall, model performance was consistent with expectations for real-world ED data and did not materially change with alternative preprocessing strategies. 

Sensitivity and robustness analyses 

Sensitivity analyses confirmed the stability of the primary findings. Re-estimating models after removing high-VIF predictors, varying Cramér’s V and VIF thresholds, or omitting SMOTE-Tomek resampling did not alter the direction or significance of key associations. BPA-related variables, including "bpa_fired_Yes," did not demonstrate meaningful predictive value in any specification. Across all analyses, demographic predictors, including race, sex, and age, did not show strong or consistent associations with naloxone distribution.

## Discussion

This study evaluated whether naloxone-related BPAs meaningfully influenced the distribution of THN in an ED. Consistent with the study hypothesis, BPA activation did not demonstrate a meaningful impact on naloxone distribution. Despite thousands of BPA firings across more than 10,000 encounters, naloxone provision remained low and unchanged. These findings indicate that, in their current form, the BPAs functioned primarily as workflow noise rather than actionable clinical signals.

In CDS, a BPA represents a signal only when it meaningfully alters clinician behavior or prompts an evidence-aligned action. In this study, clinicians appeared to rely more heavily on clinical cues such as opioid-related presenting complaints, documented substance use history, or prior naloxone administration than on the BPA itself. Although the alert fired frequently, its presence did not translate into increased naloxone distribution, a pattern characteristic of low-value alerting.

Notably, the override rate was relatively low, suggesting that clinicians did not ignore or dismiss the alert outright. Instead, clinicians engaged with the BPA but did not complete the recommended action. This pattern reflects a mechanism distinct from classic alert fatigue: The alert was acknowledged, but it did not provide information or workflow support compelling enough to change behavior.

Importantly, adjusted analyses demonstrated no significant differences in naloxone distribution across racial or ethnic groups, and BPA firing did not modify these associations. Predicted probabilities were stable across demographic subgroups, indicating that clinician responses to naloxone-related BPAs were consistent regardless of patient race or ethnicity. These findings suggest that the BPA neither introduced nor mitigated disparities in naloxone distribution.

These results have important implications for the design of harm-reduction BPAs. An alert that fires broadly or at suboptimal points in the workflow is unlikely to rise above the background noise of routine ED notifications. More precise alert logic, improved timing, and streamlined ordering pathways may help transform the BPA from noise into a meaningful clinical signal. Complementary strategies such as standing orders, automated discharge bundles, or targeted education may also be necessary to achieve meaningful improvements in naloxone distribution.

Overall, the findings demonstrate that the current naloxone-related BPA did not function as an effective clinical signal. Instead, it contributed to the background noise of ED alerts without meaningfully influencing clinician behavior. Future work should focus on redesigning BPAs to enhance relevance and usability, integrating them with broader harm-reduction strategies, and evaluating their impact prospectively to determine whether a more refined alert can serve as a true signal in opioid overdose prevention efforts.

Comparison with prior studies and strengths 

Prior evaluations of naloxone-related CDS have primarily focused on prescribing, examining whether EHR-based BPAs increase naloxone prescriptions for patients at elevated risk of opioid overdose. For example, an EHR BPA in an academic medical center significantly increased naloxone prescribing rates, and similar findings have been reported across multisite health systems where electronic advisories prompted opioid prescribers to co-prescribe naloxone during high-dose opioid encounters [10,11]. Additional work has shown that EHR prompts, combined with policy changes or education, can further increase naloxone prescribing in hospital settings [12].

Unlike prior studies that examined naloxone prescribing, this study evaluates actual naloxone distribution at the point of care, providing a more direct measure of patient-level harm-reduction impact. This distinction is important because prescribing does not guarantee that patients receive naloxone upon discharge, whereas distribution reflects a completed intervention.

Most existing BPA studies have also been conducted in academic medical centers or large health systems that differ from community EDs in workflow, staffing, and CDS adoption patterns [11]. By contrast, the present study was conducted in a community ED, offering insight into CDS performance in a setting that represents the majority of US emergency care but remains underrepresented in the CDS literature.

Prior studies have typically relied on pre/post comparisons or descriptive analyses of prescribing behavior [11]. Few have applied multivariable modeling, assessed multicollinearity, or conducted sensitivity analyses to evaluate the robustness of CDS predictors. Additionally, prior evaluations of naloxone-related CDS rarely report BPA override rates, making the present study one of the few to characterize real-world override behavior in detail. This study contributes to a more rigorous analytic approach by incorporating VIF-based variable screening, class-imbalance correction, and model performance metrics such as AUC, precision, and recall.

Together, these differences position the current study as a novel contribution that extends the existing evidence base by focusing on naloxone distribution, evaluating CDS performance in a community ED, and applying a transparent, reproducible modeling framework. Although BPA-related variables did not demonstrate meaningful predictive value, the study provides a detailed characterization of BPA behavior, including Trigger Points, Warning Names, Override Reasons, and User Follow-up Actions within real-world ED workflows [1,10].

This study has several additional strengths. It leveraged a large and diverse ED cohort, enabling robust estimation of associations across demographic and clinical subgroups. Rigorous preprocessing ensured complete BPA and naloxone documentation, reducing the risk of missing-data bias. The analytic approach incorporated multicollinearity diagnostics and VIF-guided pruning to stabilize model estimation. Examining BPA behavior at a granular level further strengthens the study’s contribution by clarifying how the alert functioned operationally, even though it did not meaningfully influence naloxone distribution [1,10].

Limitations 

Several important limitations warrant consideration. As a retrospective EHR-based analysis, the study relies on the accuracy and completeness of clinical documentation. Undocumented naloxone distribution or misclassified BPA events may have introduced nondifferential misclassification, which would likely bias the results toward the null. The cross-sectional design also precludes causal inference; associations between BPA firing and naloxone distribution should not be interpreted as causal effects. BPA logic, alert burden, and workflow integration vary across institutions, potentially limiting generalizability.

Although the analytic model adjusted for a broad set of demographic and clinical covariates, unmeasured confounders such as clinician attitudes toward harm reduction, undocumented substance use, or contextual factors influencing decision-making may still be present. Additionally, the BPA metadata available for analysis, for example, Trigger Points, Warning Names, and Override Reasons, may not fully capture the nuances of alert relevance or workflow fit, which could limit the ability to detect meaningful BPA-related effects. Finally, the study was conducted within a single health system during a period of evolving opioid-related care practices, which may limit applicability to other settings or time periods.

## Conclusions

This study set out to determine whether six naloxone-focused BPAs functioned as meaningful clinical signals or merely contributed to workflow noise. Consistent with the study hypothesis, BPA activation did not have a meaningful impact on the distribution of THN kits in this ED. Despite frequent firings, the alert did not alter clinician behavior, indicating that the BPA operated primarily as workflow noise rather than a reliable prompt for harm reduction.

Naloxone distribution was driven by clinical presentation and patient history rather than BPA exposure. Clinicians appeared to respond more strongly to overt indicators of opioid-related risk, such as opioid-related complaints, documented substance use, or prior naloxone administration, than to the alert itself. Although clinicians engaged with the BPA, the alert did not provide sufficient relevance or workflow support to influence decision-making.

These findings directly address the question posed in the study’s title. The current BPA did not rise to the level of a clinically meaningful signal. Instead, it blended into the background of routine alerts without supporting consistent distribution of THN kits. Future work should focus on refining alert logic, improving timing and usability, and integrating BPAs with broader operational strategies to ensure that CDS tools enhance, rather than dilute, harm-reduction efforts.
